# The Role of Mitochondrial Dysfunction in Idiopathic Pulmonary Fibrosis: New Perspectives for a Challenging Disease

**DOI:** 10.3390/biology12091237

**Published:** 2023-09-14

**Authors:** Juan David Cala-Garcia, German Jose Medina-Rincon, Paula Andrea Sierra-Salas, Julio Rojano, Freddy Romero

**Affiliations:** 1 Pulmonary, Critical Care and Sleep Medicine, Baylor College of Medicine, Houston, TX 77030, USA; juan.cala-garcia@bcm.edu; 2 Facultad de Medicina y Ciencias de la Salud, Universidad del Rosario, Bogota 111221, Colombia; 3 Department of Cell and Developmental Biology, University of California San Diego, La Jolla, CA 92161, USA

**Keywords:** pulmonary fibrosis, mitochondria, mitochondrial dysfunction, mitochondrial biogenesis, mitophagy

## Abstract

**Simple Summary:**

Idiopathic pulmonary fibrosis (IPF) presents a significant challenge. Despite extensive research, our understanding of its underlying causes remains limited. One intriguing hypothesis proposes that disruptions in mitochondrial functionality might play a role. Mitochondrial dysfunction is a recognized feature of the normal aging process, and parallels can be drawn to IPF, particularly within type 2 alveolar cells. This comprehensive review aims to unravel the various pathways of mitochondrial dysfunction observed in IPF. Furthermore, we explore potential therapeutic interventions designed to address this complex issue.

**Abstract:**

Mitochondrial biology has always been a relevant field in chronic diseases such as fibrosis or cancer in different organs of the human body, not to mention the strong association between mitochondrial dysfunction and aging. With the development of new technologies and the emergence of new methodologies in the last few years, the role of mitochondria in pulmonary chronic diseases such as idiopathic pulmonary fibrosis (IPF) has taken an important position in the field. With this review, we will highlight the latest advances in mitochondrial research on pulmonary fibrosis, focusing on the role of the mitochondria in the aging lung, new proposals for mechanisms that support mitochondrial dysfunction as an important cause for IPF, mitochondrial dysfunction in different cell populations of the lung, and new proposals for treatment of the disease.

## 1. Introduction

Idiopathic pulmonary fibrosis (IPF) is an interstitial lung disease characterized by progressive scarring of the lung tissue, leading to impaired respiratory function and reduced quality of life [1]. Despite extensive research, the underlying mechanisms driving the pathogenesis of IPF remain unanswered. However, emerging evidence suggests that mitochondrial dysfunction, a disruption in the normal functioning of cellular powerhouses known as mitochondria, plays a crucial role in the development and progression of IPF [2].

Mitochondria are key regulators of cellular energy production, apoptosis, and redox balance, and their dysfunction has been associated with implications in various diseases. Perturbed mitochondrial function contributes to altered metabolic processes, increased oxidative stress, and impaired cell survival mechanisms, all of which are hallmark features observed in IPF [2,3,4]. Understanding the relationship between IPF and mitochondrial dysfunction is crucial for identifying novel therapeutic targets and developing targeted interventions. In this review, we aim to explore the current literature surrounding IPF and mitochondrial dysfunction by highlighting the underlying mechanisms, the latest advances in the field, and possible therapeutic interventions that could be addressed soon for IPF patients.

## 2. The Aging Lung

Aging represents a natural process in life due to exposure to a variety of factors such as molecular stressors, infectious agents, allergens, pollution, radiation, and environmental exposure that lead to the development of molecular changes. During late-life decline, these changes will produce a gradual deterioration of the tissue that can be represented by structural and functional changes, where the tissue becomes more vulnerable to disease and perpetuation of its health [5]. Humans reach full pulmonary function and maturation between the ages of 20 and 25 years [6]. However, after reaching the age of 65, pulmonary function starts declining progressively. This leads to impaired vital functions and reduced ability to adapt to the environment. This manifests as metabolic dysregulation, reprogramming, and dysfunction [7]. 

### 2.1. Mitochondrial Function in the Normal Lung

Mitochondria are one of the most important organelles in eukaryotic cells, with the main function of generating most of the energy of a cell [8]. Mitochondria not only produce cellular energy through oxidative phosphorylation, but also have critical cell functions such as regulating calcium homeostasis as well as programmed cellular death [9,10]. In the lung, mitochondria might have an additional role that is key for multicellular species, which is the ability to sense O_2_ levels at the cell. By consuming O_2_ in the electron transport chain, mitochondria are the organelle with the highest oxygen consumption, reducing oxygen intracellular tension and generating an O_2_ gradient from the extracellular to the intracellular space [11]. In hypoxia, it has been demonstrated that a triggering vasoconstriction response by artery-smooth muscle cells [12], a response that could be led by a decrease in mitochondrial oxidative phosphorylation, is a processing that leads to a leak of reactive oxygen species (ROS) to the cytosol. The increase of ROS in the intracellular space generates a release of intracellular calcium that eventually leads to smooth muscle cell contraction and later, arterial vasoconstriction [13]. In the pulmonary vasculature, vasoconstriction in hypoxia is a homeostatic mechanism where blood is directed to the lung segments with better oxygenation, optimized ventilation/perfusion, as well as oxygen delivery to the whole body [14].

If hypoxia persists, the increase of ROS can lead to a leak of mitochondrial DNA (mtDNA). mtDNA is a recognized damage associate molecular pattern (DAMP) that triggers the activation of NALP3 and TLR9 and further activation of a downstream proinflammatory response [11]. mtDNA release not only progresses to a proinflammatory response, but can also be linked to further cellular stress responses such as mitophagy and programmed cell death, features seen in the aging lung and IPF [15]. 

### 2.2. Adjustments in Respiratory Mechanics, Flow, and Lung Volumes

With aging, there are several changes in the extrapulmonary structures that will impact the respiratory mechanics, and therefore alter the lung’s normal function. Initially, there is a modification in the thoracic shape due to a loss of the vertebral body height that narrows the intervertebral spaces generating a collapse of the vertebral bodies that causes a curvature of the spine, known as kyphosis. This tendency results in a reduction of the intercostal space, leading to a smaller chest cavity and decreased compliance of the chest wall. Consequently, the rib cage becomes stiffer, primarily due to a decrease in the thickness of the parietal muscles. As a result, there is a notable decrease in the strength of the inspiratory and expiratory muscles [16,17]. Furthermore, in the aging lung there is a decrease in elastic recoil pressure that affects the expiratory flows and lung volumes. This elastic recoil represents an important factor in the depletion of surface tensions that generates an alteration in the diameter of the alveoli and alters the normal mechanisms of breathing [18]. The inability to ventilate the increasing demands adequately will generate a depletion in the forced vital capacity (FVC) and the forced expiratory volume in 1 s (FEV1). This rapid detriment creates a flow volume loop in which the expiratory flow decreases, while the residual volume (RV) increases [17,19]. The increased RV will also decrease the ability to clear mucus from the lungs because of a reduced cough strength. Coughing requires a high expiratory flow and rapid contraction of inspiratory muscles to intake large amounts of tidal volume to maintain a high expiration flow. As mentioned before, there is a decrease in the strength of respiratory muscles, which means efforts to generate a cough impulse will be insufficient [16]. 

### 2.3. Cellular Changes in the Aging Lung

Aging is an extraordinary process that represents a multifactorial change in different aspects of life and can involve genome failures, signaling dysfunction, organelle compromise, cell communication abnormalities, and progressive loss of cellular functions [5]. When aging, the lung experiences structural changes and architectural changes. The respiratory epithelium, or alveolar epithelium, is a vital barrier between the outer environment and internal tissues and works as the first line of defense against pathogens, particulates, and exterior materials. Two distinct cell types, alveolar epithelial type 1 cells (ATI) and alveolar epithelial type 2 cells (ATII), mainly compose the alveolar epithelium. ATIs primarily facilitate gas exchange within the alveolar unit, while ATIIs are responsible for producing surfactants to maintain fluid balance. Additionally, ATIIs also serve as progenitor cells for ATIs [5].

Aging progressively deteriorates the epithelium leading to a continuous loss of alveolar surface area as the result of the destruction of both ATIs and ATIIs. Additionally, aging induces endoplasmic reticulum stress, leading to the accumulation of immune cells and the establishment of a cytokine-rich environment. These changes promote fibroblast proliferation, whose main function is to support the growth of epithelial cells. The activated epithelial cells and fibroblast participate in the secretion of extracellular matrix proteins, altering the production and secretion of collagen. Consequently, this process disrupts the organization of collagen fibers, resulting in alveolar duct dilatation and the loss of elasticity in the airspace [5]. The excessive proliferation of fibroblasts, deposition of fibrotic tissue, and elevated levels of proinflammatory cytokines collectively create a state of inflammatory aging. This condition is characterized by an upregulation in the production of proinflammatory interleukins, including IL-1B, IL-6, TNF, TGF-beta, and others. The sustained proliferation of these factors contributes to the dysregulation of cytokine production, which in turn leads to immune inflammation and increases susceptibility to infections [20]. The secretion of cytokines by the pulmonary immune system has an impact on surrounding leukocytes and induces the upregulation of toll-like receptors. This results in a reduction of alveolar macrophages and peribranchial macrophages, leading to multiple functional defects. These defects include compromised phagocytic activity and an impaired response to signaling, which are directly associated with immunosenescence. Consequently, these phenomena end with an increased susceptibility to infections, exacerbated damage, and slow the healing process [5].

Another important factor involved in lung aging is the organelle compromise directly related to mitochondrial abnormalities. Mitochondria are known to be responsible for energy production through the process of oxidative phosphorylation, aided by the process of biogenesis. Biogenesis represents the production of additional mitochondria to extend cellular energy production, but the process is affected by aging due to the decrease of activators such as PPAHy coactivator 1-alpha and 1-beta. The deficiency in mitochondrial biogenesis also predisposes mitophagy to occur. Mitophagy represents the response that targets mitochondria to regulate the number of organelles to match cellular energy needs and remove the ones that are damaged or dysfunctional and may cause more cellular stress [21,22]. With aging, the process of mitophagy may be affected. PTEN Induced Kinase 1 (PINK1) is a molecule that regulates mitophagy and damaged mitochondria by regulating mitochondrial membrane depolarization as well as marking dysfunctional mitochondria [22]. With age, recent findings establish a decrease of PINK1 expression as well as an increase of endoplasmic reticulum stress [7]. Alternatively, both intrinsic and extrinsic factors contribute to cellular pathways that mtDNA damage. This damage negatively affects transcription, reduces transmembrane potential, and elevates levels of ROS. Oxidative damage occurs, rendering the cells to be more susceptible to injury and further dysfunction. In addition, these processes impair the capacity of mitochondria to transport electrons, leaving them in a nonfunctional state [23]. 

## 3. Mitochondria and Idiopathic Pulmonary Fibrosis

Mitochondrial function has recently become a target of study given its potential role in certain lung disease states related to the cells of the interstitium. IPF, although still considered irreversible, despite having rather slow progress in treatment, has had certain breakthroughs regarding its potential etiology, which appear to include mitochondrial dysfunction. An important process to credit regarding the normal maintenance of cellular and particularly mitochondrial function regulation is mitophagy. Like autophagy, mitophagy is the process by which profoundly damaged mitochondria undergo a machinery-heavy process in which lysosomal enzymes will eventually destroy and allow for the recycling of the damaged organelle which leads the reinstitution of cellular and mitochondrial homeostasis [21]. This process has recently shed light on certain states of both health and disease, diseases such as IPF. It is key to mention that there is high-quality evidence that points to the fact that tissue with higher rates of mtDNA damage and mutations are associated with pathological states, shown particularly in the aging lung [24,25,26].

**Figure 1:**
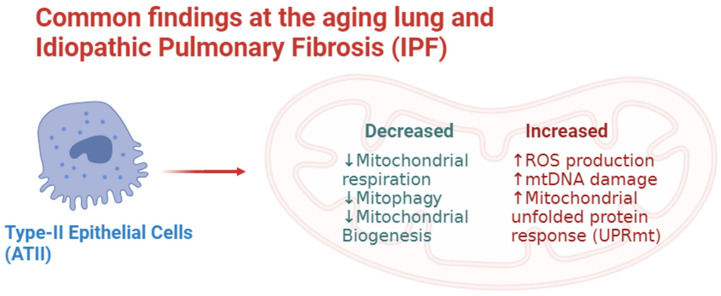
Common findings at the aging lung and idiopathic pulmonary fibrosis.

The first key finding that merits mentioning is the previously exposed role of mitochondria in the aging lung. It is now clear that these age-related processes may precipitate IPF and its progression. Moreover, in genetically susceptible patients, this association may be more pronounced or reach clinical significance. This association appears to have its origin in senescent cells in the interstitium, particularly, fibroblasts and myofibroblasts which are responsible for the production of extracellular matrix proteins [27,28]. This process, often regarded as the senescence-associated secretory phenotype or SASP has recently been the subject of study given the high association of atypical senescent myofibroblasts and a high extracellular matrix protein concentration [27]. A second finding that adds up to the role of mitochondria in IPF is the decreased effectiveness of mitochondria in aging cells. Several models have found a relationship between aging mitochondria and decreased ATP production [29]. More so, these older mitochondria when producing less ATP generates increasing amounts of ROS at the same time [30]. It is worth mentioning that a potentially additive effect to this phenomenon is the altered structure of mitochondria in older cells; these structural abnormalities include the destruction of inner membranes, reduction or absence of cristae, and overall mitochondrial enlargement [31]. Common findings between ATII cells at the aging lung and IPF are illustrated in Figure 1.

A finding that has caused some controversy regarding these processes are the models altering mitochondrial function and the genetic expression of electron transport chain proteins that have been associated with an increase [32,33]. Similar to what happens with autophagy and cellular apoptosis, there appears to be a relationship with the magnitude of mitochondrial impairment, meaning that when there is mild to moderate mitochondrial stress, longevity might be increased, yet severe damage to mitochondrial function will likely shorten the lifespan [34]. Part of this phenomenon is explained by the mitohormesis paradigm, a recently proposed hypothesis suggesting that temporary mitochondrial stress, which is not severe enough to promote apoptosis and cellular damage, may result in several nuclear and cytosolic changes through which the cell will reprogram metabolic and biochemical pathways in a way that may result beneficially towards survival and may also determine longevity [35].

The aforementioned mechanisms account for part of the histopathological findings in IPF; nonetheless, there have been other mitochondrial dysfunction processes found to contribute to the development and progression of IPF in other cells such as type II alveolar epithelial cells. In this cell type, mitochondrial accumulation has been found both in the aging lungs and in lungs with IPF [36,37]. In the latter, an altered surfactant structure has been found to accelerate aging [37,38]. Similar findings such as those concerning fibroblasts have been found, particularly regarding protein expression. The most studied proteins found in this cell type are those related to apoptosis and TFG-beta signaling: SIRT3 and PINK1 [39]. 

A low expression of PINK1 has been associated with increased apoptosis, increased susceptibility to mitochondrial damage, stress, and to lung fibrosis, through what appears to be a TFG-beta-mediated process [39,40,41]. Furthermore, this protein has also been linked to neurodegeneration in the elderly [42,43], a fact that might shed light on the role of this protein in mitochondrial homeostasis in non-mitotic tissues. On the other hand, SIRT3 (Sirtuin 3) has recently gained interest due to its potential role in mitochondrial damage in aging tissues. This protein has been associated with an increased susceptibility of the lung to injury and making it prone to fibrosis by promoting fibroblast to myofibroblast transformation by suppressing TGF-beta signaling [44,45]. Bleomycin exposure has shown increased lung fibrosis due to SIRT3 downregulation which promotes susceptibility [46,47]. This finding has been replicated in aging mice and asbestos exposure models [48]. Interestingly, the SIRT family has not only been associated in a said way with IPF, but another important pathway that appears to modulate mitochondrial instability, which is further susceptible to developing IPF-related histopathological changes, also coming from epigenetic modifications. In normal conditions, SIRT1 acts as a suppressant of the previously mentioned SASP by stimulating the deacetylation of histones in the promoter regions of secretory proteins [49].

Another pathway through which SIRT3 appears to exert its functions in normal cellular architecture and function is by modulating the acetylation of 8-oxo guanine-DNA glycosylase-1 (OGG1). OGG1 is an enzyme highly active in situations of ROS-induced mtDNA damage and stress. It is primarily responsible for cleaving oxidized guanines through hydrolyzation [50]. This crucial role of the SIRT3–OGG1 interaction has been shown in the setting of an early-set aging phenotype, which is associated with a high rate of mutations in mtDNA and has been shown to cause polymerase gamma mtDNA proofreading dysfunction [51]. All these findings in the SIRT3 function point towards its relevance as a susceptibility factor for the development of IPF.

It should be noted that chronic exposure to both low levels of oxygen and high levels of CO_2_ have been shown to induce mitochondrial stress in both epithelial alveolar cells, and cells surrounding the pulmonary parenchyma such as fibroblasts; such findings have been shown to prolong ROS-mediated injury to healthy tissues and to induce fibroblast transformation to SASP cells such as myofibroblasts [52,53].

PINK1 and SIRT3 are not the only genes associated with mitochondrial dysfunction in IPF. It has been demonstrated in animal models that insufficient mitophagy in the fibroblastic foci could potentially be explained by a PARK2-mediated platelet-derived growth factor receptor signaling pathway, which leads to an enhanced myofibroblast differentiation and proliferation [54]. When carefully reviewing the myofibroblast gene expression, the authors also showed that PARK2 dominantly regulates the myofibroblast differentiation when compared to PINK1 [54].

Alveolar epithelial cells or fibroblasts/myofibroblasts are not the only cells that present mitochondrial dysfunction in the fibrotic lung. Macrophages, especially in environmental-associated interstitial diseases such as silicosis or asbestosis, also have an important cellular bioenergetics dysfunction [29]. Akt1 is a threonine-protein kinase associated with the production of ROS [55]. Akt1-deficient murine models exhibited reduced mitophagy and decreased expression of transforming growth factor–beta1 (TGF-β1), particularly noticeable in alveolar macrophages. Upon asbestos exposure, this phenotype was protected from pulmonary fibrosis through enhanced mitophagy activation [56]. In addition, primary alveolar macrophages from IPF subjects show a decrease in oxidative phosphorilation gene expression and an increase of mitochondrial ROS [57]. One of the main drivers of an increase of mitochondrial ROS is the enzyme NADPH oxidase-4 (NOX4), which on the macrophages is not only involved in higher ROS, but also induces mitochondrial biogenesis on them, when this phenomenon is usually downregulated in the other cell populations [29]. Alveolar macrophages collected from subjects with asbestosis presented with a high expression of NOX4, where it is thought to confer a profibrotic environment [58]. On fibroblasts, NOX4 is associated with a decrease on mitochondrial biogenesis in IPF subjects due to its downregulation of both nuclear factor erythroid-derived 2-like 2 (NRF2) and mitochondrial transcription factor A (TFAM), both being transcription factors that are associated with high mitochondrial biogenesis [59]. Finally, NOX4 has an interesting role in the transformation from fibroblasts to myofibroblasts in IPF subjects, where it has been proposed that the increase of ROS due to NOX4 overactivity could be a trigger factor for differentiation for myofibroblasts in the IPF lung [60].

Table 1: Mitochondrial alterations from different cell populations in the IPF lung.

## 4. Newest Findings on Research for Mitochondrial Dysfunction

Proper mitochondrial function is linked to cellular senescence regulation, adequate communication with other organelles such as the endoplasmic reticulum, and regulation of mtROS [61]. Discoveries have taken place in the past years that have helped better understand the role of mitochondria in idiopathic pulmonary fibrosis (IPF).

Cellular senescence is a key phenomenon in IPF [62], but in this complex disease they have proven to have different cellular components exhibiting this feature [61]. New technologies such as single-cell RNA sequencing (scRNAseq) have proven to be effective to understand the role of each cell in the disease, as well as being a discovery agent for new cells specific for certain diseases [63]. By using scRNAseq on human lung samples, it has been demonstrated that ATII cells present with higher senescence markers (such as CDKN1A/p21, CDKN2A/p16, TP53, MDM2, and CCND1) were higher in IPF lung samples when compared to controls, as well as demonstrating higher activation of senescence-related pathways such as oxidative phosphorylation, mitochondrial dysfunction, and the sirtuin signaling pathway [64]. The authors later validated these findings with a novel mouse model, in which they created Sftpc^CreER^, Sin3^af/f^, and Rosa^mTmG^ mice with conditional Sin3a loss of AT2 cells that led to a p53 and p21-dependent senescence state in AT2 cells [64]. In addition, the major scRNAseq data set published on IPF also validated the presence of senescence markers, mainly on AT2 cells as well as in a new disease-specific cell that expressed both epithelial and mesenchymal markers that could be important for the activation in the epithelial to mesenchymal transition (EMT) process in IPF [65].

Another tool that has come up in recent years to assess mitochondrial functioning is the Agilent Seahorse XF Analyzer platform, which allows for the measuring of the mitochondrial oxygen consumption rate (OCR) and extracellular acidification rate (ECAR) [66]. By injecting different drugs, several parameters associated with mitochondrial functioning such as basal respiration, ATP production, proton leak, maximal respiration, and spare respiratory capacity will be assessed during the assay [67]. This experiment can be valuable for the study of aging and fibrosis in different organs, and lung fibrosis has not been the exception. It has been shown with this assay on animal models that the oxygen consumption rate (OCR) is decreased in old ATII cells in comparison to ATII obtained from 3-month-old mice, and, when an endoplasmic reticulum (ER) stressor such as tunicamycin was administered in old and young ATII cells, the same decrease in OCR was also observed [39]. In the same article, with different methods, the authors associated the decrease in OCR in aging ATII cells with the low expression of PINK1. By knocking down PINK1 on mice ATII cells, the authors detected an increase in profibrotic factors as well as observing dysmorphic and dysfunctional mitochondria in the ATIIs that were more vulnerable to apoptosis and the development of lung fibrosis [39].

In another paper, the authors saw that the knocking out of PINK1 in mice would lead to an increase of mitochondrial DNA (mtDNA) oxidation in lung tissue, a finding that was also observed in human cells from IPF lung samples, where not only PINK1 expression is lower when compared to control samples, but also associated to an increase of mtDNA oxidation [68]. The importance of mtDNA oxidation in human samples was further demonstrated by treated precision-cut lung slices (PCLS) from control lungs with this component for 24 h. In this experiment, the authors not only saw an increase in TGF-beta expression but also saw that this response was higher in PCLS from older subjects [68]. 

Furthermore, an association between PINK1, ER stress, and mitochondrial dysfunction was proven recently, both in animal models as well as human cells [69]. Integrated stress response (ISR) corresponds to the system that guides the communication of the mitochondria with the cell nuclei and other organelles, a process associated with the activated transcription factor family (ATF) [70]. ATF3 overexpression decreases the activity of the PINK1 promoter in human lung cell lines and was associated with altering mitochondrial homeostasis. In addition, it was shown that ATF3 expression is higher in bleomycin-injured ATII cells as well as IPF ATII human cells [71]. Nevertheless, the association of mitochondrial dysfunction with ER stress was not clear until a research group demonstrated that in ATII mice cells, an unfolded protein response in the ER (UPR^ER^) generates a similar response at the mitochondrial level through an ATF4-dependent pathway in aged mice with bleomycin-induced lung injury as well as human cells from subjects with IPF [72]. All these findings would not have been possible without utilizing novel techniques such as the Agilent Seahorse XF Analyzer platform for assessing OCR and ECAR, animal models, and the implementation of translational medicine models for the obtention of human ATII cells. 

Although mitochondrial dysfunction has been relevant in the role of ATII cells in IPF, the evaluation of mitochondrial respiration in fibroblasts on IPF has also revealed new insights for IPF pathogenesis as well as possible therapeutic interventions. Caporarello et al. associated the dysfunction of the transcription factor peroxisome proliferator-activated receptor gamma co-activator 1-alpha (PGC1α) in IPF fibroblasts, finding a decrease in OCR when silencing PGC1α with siRNA in control fibroblasts, and an improvement on mitochondrial mass and OCR on IPF fibroblasts when transfecting a PGC1α-vector [73]. 

## 5. New Treatment Perspectives

Here, we have reviewed the role of mitochondrial dysfunction in IPF; nevertheless, all this knowledge should be used not only to understand better the physiopathology of the disease but also to propose and envision new therapeutic targets for the improvement of the quality of life of IPF patients.

Although studied for scleroderma (SSc), an autoimmune disease associated with multiorgan fibrosis and vasculopathy [74], a novel fluorinated synthetic honokiol analog called hexafluoro was used to increase cellular SIRT3 in fibroblasts, which led to a decrease in TGF-beta signaling and mtROS [75]. In the Parkinson’s disease field, by using mass spectrometry some authors have suggested that targeting the deletion of de-ubiquitinase USP30 could serve as a promising therapeutic strategy to promote mitophagy and increase substrate ubiquitination [76,77]. More related to the fibrotic lung, it has been proposed that molecules such as MitoQ, a mitochondrial antioxidant, acts as an ROS scavenger within the mitochondria, decreasing TGF-beta and NOX4 expression in the fibroblast of IPF patients, attenuating inflammation, and collagen deposition [78]. 

Finally, there is some evidence that suggests that AMPK activators (such as metformin) in human myofibroblasts from the lungs of humans with IPF could decrease its fibrotic activity, and improve mitochondrial biogenesis, as well as their sensitivity to apoptosis if lung injury is present [79]. 

## 6. Conclusions

In conclusion, the growing body of evidence supports the significant role of mitochondrial dysfunction in the pathogenesis of idiopathic pulmonary fibrosis (IPF). The disrupted mitochondrial function contributes to the metabolic dysregulation, oxidative stress, and impaired cell survival mechanisms observed in IPF. Understanding this intricate relationship opens new avenues for targeted therapies aimed at restoring mitochondrial homeostasis. By targeting mitochondrial dysfunction, it may be possible to mitigate the progression of IPF and improve patient outcomes. Further research is warranted to elucidate the precise molecular mechanisms underlying the interaction between IPF and mitochondrial dysfunction. These insights hold promise for the development of innovative treatment strategies that may revolutionize the management of IPF in the future.

## Figures and Tables

**Table 1:** Illustrates different cells and its mitochondrial function in the IPF lung.

Cellular Type	Mitochondrial Dysfunction Features
ATII [29,36]	Increased: ROS production. Release of mtDNA. Senescence markers (CDKN1A/p21, CDKN2A/p16, TP53, MDM2, and CCND1). Decreased: Mitophagy.
Fibroblasts [59]	Increased: ROS production. Senescence. Decreased: Mitophagy. Programmed cell death.
Myofibroblasts [54,60]	Increased: Enhanced differentiation and proliferation. Progression of the fibroblastic foci on IPF. ROS production. Decreased: Mitophagy.
Macrophages [57,58]	Increased: ROS production. Mitophagy. Mitochondrial biogenesis. Decreased: Oxidative phosphorylation.

## Data Availability

No new data were created or analyzed in this study. Data sharing is not applicable to this article.
